# Chemical shift imaging: An indispensable tool in diagnosing musculoskeletal pathologies

**DOI:** 10.4102/sajr.v25i1.2061

**Published:** 2021-05-06

**Authors:** Vandana Jahanvi, Abhimanyu Kelkar

**Affiliations:** 1 Department of Radiodiagnosis, Bharati Vidyapeeth Medical College, Pune, India

**Keywords:** MRI, chemical shift imaging, musculoskeletal, in-phase imaging, opposed-phase imaging, bone marrow

## Abstract

Chemical shift imaging (CSI) is an important fat-suppression technique in magnetic resonance imaging (MRI); it is used routinely in abdominal imaging to detect the presence of intralesional fat. Its utility in musculoskeletal imaging has recently gained interest as a technique that is complementary to routine imaging. It is believed to aid in diagnosing and differentiating various osseous pathologies. This review describes the role of CSI as an imaging technique for diagnosing various osseous and periarticular pathologies in different clinical scenarios.

## Introduction

Magnetic resonance imaging (MRI) is a sensitive imaging modality for the evaluation of bony lesions. It is frequently used after routine radiographs for the diagnosis of musculoskeletal disorders. The loss of normal fatty marrow can be the result of many pathologies, which can be focal or diffuse, benign or malignant. The diagnosis and differentiation of various marrow pathologies can be challenging and require the use of various sequences other than the routine T1- and T2-weighted images, which include fat-suppression sequences, diffusion-weighted imaging, MR spectroscopy and post-contrast imaging. Various fat-suppression techniques are available to aid in musculoskeletal imaging. These sequences include frequency-selective fat saturation techniques, inversion recovery, hybrid technique, chemical shift imaging (CSI) and the related Dixon-based approach.^1^

Different techniques have their advantages and disadvantages, which make them suitable in various settings for different protocols and varying magnet strengths. Chemical shift imaging with in-phase (IP) and opposed-phase (OP) imaging is a routinely used sequence for the detection and characterisation of hepatic, renal and adrenal lesions.^2^ The use of CSI in the diagnosis of localised and diffuse bone marrow lesions has recently become a topic of keen interest. However, there are still only a limited number of studies that highlight the importance of this technique in the assessment of musculoskeletal disorders. In this review, we aim to illustrate the utility of CSI as a problem-solving tool for the identification of various osseous and periarticular pathologies.

## Chemical shift imaging physics

The main principle behind CSI is that it utilises the difference in the precession frequencies of fat and water molecules. Compared to water molecules, fat molecules have higher shielding of protons.^3^ Thus, protons in fat molecules precess at a slightly lower frequency than protons in water.^4^ This frequency difference is known as the *chemical shift effect*. This effect is exploited during IP and OP imaging in CSI. The difference in phase between the images acquired at different echo times (TE) is the basis of OP imaging.^5,6,7,8,9^ The phase is the angle of magnetisation vector in the transverse plane. Because fat and water protons have different precession frequencies, the phase of these protons, with respect to each other, changes a few milliseconds after the initial excitation.^10^

**FIGURE 1:**
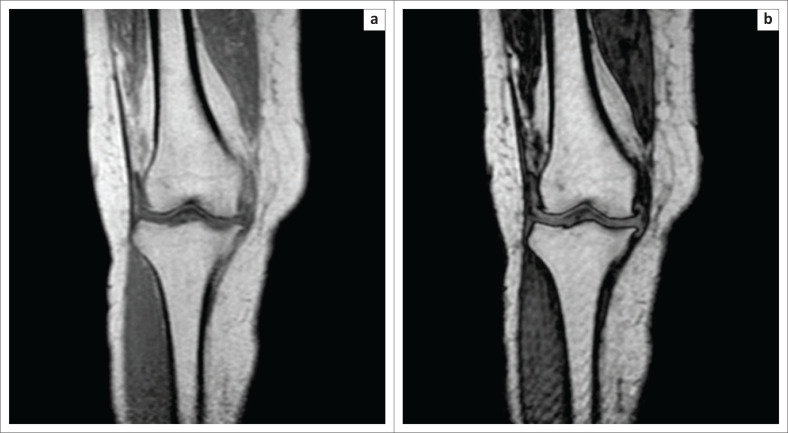
Sequence comparison between in-phase (a) and opposed-phase (b) images. The opposed-phase image (b) is easily identified, owing to the India ink artefact that is present at the interface of the different anatomical structures (observed as a signal void [hypointensity]).

Immediately after the excitation, fat and water protons are in-phase with each other. However, because fat protons precess at a slower rate compared to water protons, after a few milliseconds, the phase difference between them becomes 180°, that is, they become out of phase. A few milliseconds after the out-of-phase state, water protons complete a 360° rotation relative to fat protons; thus, they are again in-phase. The signals from the fat and water molecules add onto each other in the IP images. However, in the OP images, the signal is the difference between the signals from the fat and water molecules. Therefore, OP imaging reduces or suppresses the signal from fatty tissue.^10^ At the margins between fat and non-fatty tissue, signals from adjacent water and lipids can be equal. This results in a signal void at the interface of fatty–normal tissue, which is termed the *India ink artefact* or *black boundary artefact* (Figure 1).^8^ The OP images can be recognised on account of this feature.

Because precession frequency and magnetic field strength are related, the TE at which the IP and OP images are acquired depends on the magnetic field strength. Using the 1.5T scanner installed at our institute, the IP images are acquired at 4.6 ms and the OP images at 2.3 ms.

Opposed-phase imaging is simple and quick. Chemical shift imaging is mainly able to demonstrate microscopic or intracellular fat. The OP images show a reduced signal from voxels that contain both fat and water. However, it does not suppress the macroscopic fat. The ability to demonstrate a small amount of fat and fat–water mixtures is the strongest advantage of this technique.^5,7,8,9^ On the other hand, imaging techniques such as short tau inversion recovery (STIR) and spectral attenuated inversion recovery (SPAIR) suppress only the macroscopic fat. Because it is dependent on the phase difference between fat and water protons, this imaging technique is independent of static field heterogeneity.

## The role of opposed-phase imaging in various osseous and periarticular pathologies

### Marrow lesions

The maturation of normal marrow involves the replacement of red marrow with yellow marrow, which progresses in a centripetal fashion with a near-total loss of red marrow by adulthood.^1^ The reversal of marrow maturation in response to increased hematopoietic demand results in marrow reconversion. In adults, the chemical composition of yellow marrow is primarily fat, which is approximately 80% fat and 15% water. In contrast, red marrow contains approximately equal portions of fat and water (approximately 40% each).^11^

Owing to this division in chemical composition, MR can sensitively detect marrow changes.^12^ Fat-suppressed proton density-weighted images may show focal islands of reconverted red marrow, which may mimic neoplastic lesions.^13^ This occurs because red marrow exhibits incomplete suppression on routine fat-suppression sequences compared to yellow marrow. In CSI, normal and reconverted marrow demonstrate a greater than 20% signal loss on OP compared to IP images.^9^ This reduction in signal intensity is calculated by placing a region of interest (ROI) in the involved area on the IP and corresponding OP images. To determine if there is signal suppression, we determine the signal intensity ratio. This is calculated by dividing the signal intensity value at the ROI on the OP by the signal intensity at the ROI on the IP images.

Red marrow islands in children appear as hyperintense areas on SPAIR and STIR images and may be confused with marrow oedema or marrow infiltrative disorders. Because red marrow demonstrates signal suppression, this property is utilised at CSI to differentiate it from other sinister pathologies; since they do not contain microscopic fat, there is no signal suppression on OP images. Similarly, in adult patients with marrow reconversion, CSI may help us to differentiate this entity from marrow infiltrative disorders like leukaemia. This confusion arises because in both marrow infiltrative disorders and marrow reconversion, bone marrow appears hypointense on T1W images (in contrast to T1W hyperintensity in normal yellow marrow) and hyperintense on routine fat-suppressed images.

**FIGURE 2:**
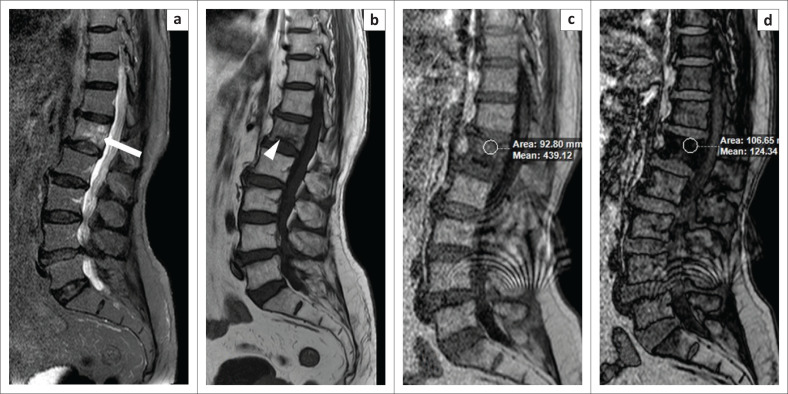
A 77-year-old man experienced back pain for two weeks. Magnetic resonance imaging revealed marrow oedema (white arrow) involving the L1 vertebra on the T2W spectral attenuated inversion recovery (a) image. A horizontal fracture line (white arrowhead) was evident on the T1W image (b). By comparing the in-phase (c) and opposed-phase (d) images, it was determined that the decrease in signal intensity on the opposed-phase image was 71% (>20%), which ruled out an underlying small metastatic lesion. The final interpreted diagnosis was that of an osteoporotic compression fracture.

**FIGURE 3:**
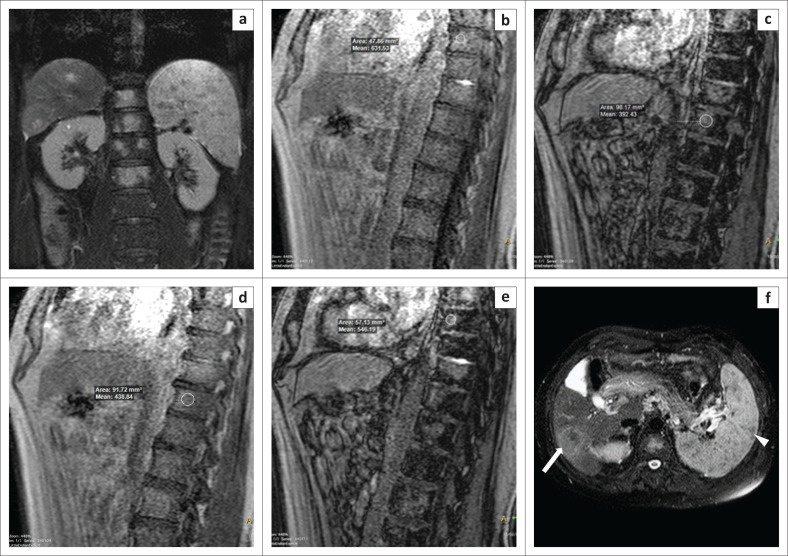
Vertebral metastases. A 67-year-old man complained of back pain for 1 month. Magnetic resonance imaging showed multiple areas of abnormal signal within the thoracolumbar vertebrae, which were hyperintense on coronal T2W spectral attenuated inversion recovery (a) images. By comparing the in-phase (b, d) and opposed-phase (c, e) images, it was determined that the decrease in signal intensity on the opposed-phase images was 13% and 10% (<20%) at two different vertebral levels, which indicated the probability of metastases. Upon screening the abdomen, it was observed that the patient also had multiple lesions in the spleen (white arrowhead) and a lesion in the liver within segment VI (white arrow), as seen on the axial T2W spectral attenuated inversion recovery image of the abdomen (f). We therefore concluded that the vertebral lesions were metastases. However, the patient did not undergo a biopsy.

Chemical shift imaging may also help us to differentiate neoplastic versus non-neoplastic lesions depending on the amount of fat suppression at OP imaging. If the signal loss is less than 20% on OP images, this indicates replacement of normal fat, which could be the result of the presence of benign or malignant lesions in the marrow (Figures 2 and 3).^14^ However, in a study on 50 patients with intermediate bone marrow lesions in the pelvis,^15^ it was determined that the criterion of less than 20% raises the suspicion for a malignant lesion; this approach allowed for the correct diagnosis of 27/27 malignant lesions and 14/23 benign lesions, which yielded 100% sensitivity, 61% specificity, 75% positive predictive value, 100% negative predictive value and 82% accuracy.

Healed and active neoplastic lesions after chemoradiotherapy (e.g. metastasis or myeloma) may appear similar when conventional MR imaging is used, which may cause confusion. Healed lesions exhibit intra- or perilesional fatty signal intensity on CSI. By identifying otherwise inconspicuous fat on OP imaging, one can avoid misdiagnosing these lesions as active lesions and correctly guide patient management.^1^

### Fractures

**FIGURE 4:**
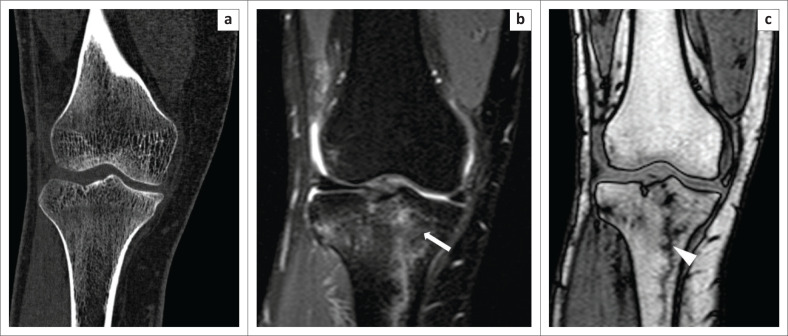
Trabecular fracture, better demonstrated by chemical shift imaging. Computed tomography (CT) and magnetic resonance imaging of a patient with trauma to the knee. No obvious fracture line was observed at CT (a). On the Proton-Density-Weighted spectral attenuated inversion recovery image (b), an irregular vertical hyperintensity was present with associated marrow oedema (white arrow). On the opposed-phase image (c), a clear irregular zigzag vertical fracture (white arrowhead) was seen.

Trabecular fractures may be masked on fat-suppressing techniques (such as STIR) owing to marrow oedema, but can be identified at OP imaging. It is believed that OP imaging is more sensitive than conventional PD-weighted (Proton-Density) and T1-weighted imaging in demonstrating trabecular distortion and the extent of microfractures (Figure 4).^1^ In addition, CSI can differentiate between benign and pathologic fractures by the loss of signal on OP imaging.^16,17^ In pathologic fractures, the signal loss is believed to be less than 20%.

### Subchondral degenerative changes and -epi-metaphyseal lesions

**FIGURE 5:**
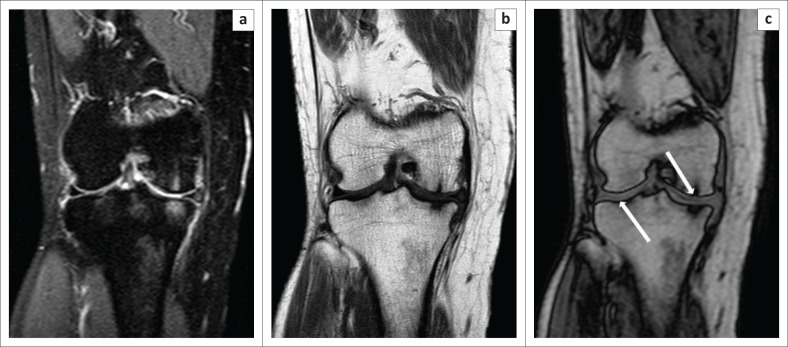
Degenerative changes. A 49-year-old female presented with a history of chronic right knee pain. The Proton-Density-Weighted spectral attenuated inversion recovery image (a) showed areas of subchondral hyperintensity, which was suggestive of marrow oedema. On the T1W (b) image, subchondral marrow hypointensity was observed. On the opposed-phase image (c), thinning of the cartilage (white arrows) at the site of the subchondral signal change was observed, which was consistent with changes of osteoarthritis.

Cartilaginous end-plate contour changes are observed on OP images with good differentiation of cartilage from the bone (Figure 5).

**FIGURE 6:**
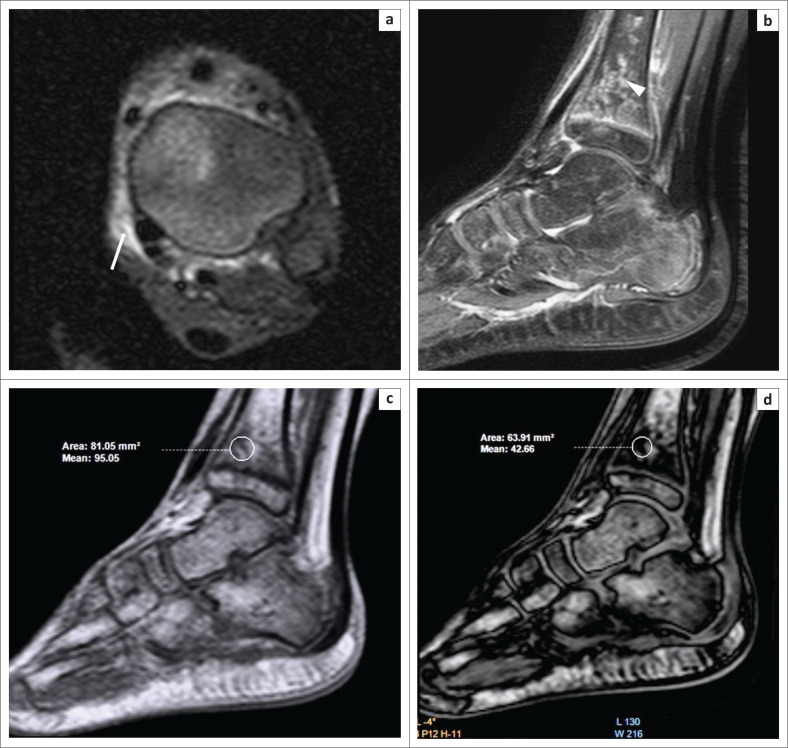
Differentiating islands of red marrow from other sinister pathology. A 10-year-old boy presented with a 10-day history of pain and swelling over the left ankle. Magnetic resonance imaging showed soft tissue oedema (white arrow) over the medial aspect of the tibia on the axial Proton-Density-Weighted (PDW) spectral attenuated inversion recovery (SPAIR) image (a). Areas of hyperintensity were observed on PDW SPAIR (b) within the epiphysis and metaphysis of the distal tibia (white arrowhead). On comparing the in-phase (c) and opposed-phase (d) images, it was determined that the decrease in signal intensity on the opposed-phase images was 55% (>20%), which suggested the presence of red marrow and ruled out any sinister intraosseous pathological lesions such as osteomyelitis.

Subcortical cysts or geodes may sometimes mimic more sinister epiphyseal lytic lesions, including giant cell tumours and chondroblastoma. Moreover, in children, islands of red marrow (Figure 6) at the epi-metaphysis may be confused with marrow pathologies. The presence of fat within lesions helps to confirm benign aetiology.

### Avascular necrosis and sclerotic foci

**FIGURE 7:**
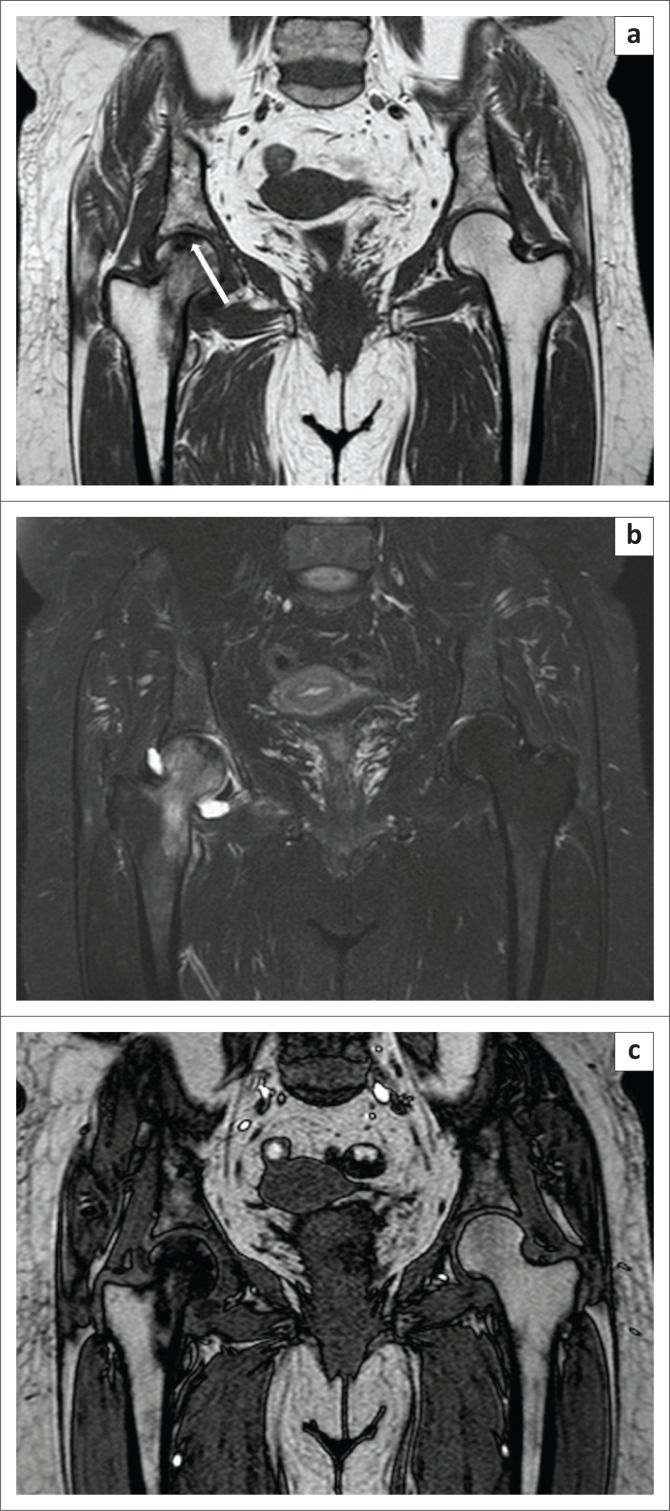
Avascular necrosis of the right femoral head. A 28-year-old man presented with pain in the right hip over a period of 3 months. The T1W image (a) showed subchondral hypointensity (white arrow) and contour irregularity of the right femoral head. On the Proton-Density-Weighted spectral attenuated inversion recovery (b) image, hyperintensity was observed within the involved bone, which was suggestive of marrow oedema. The opposed-phase image (c) revealed a clear demarcation of the infarcted zone.

**FIGURE 8:**
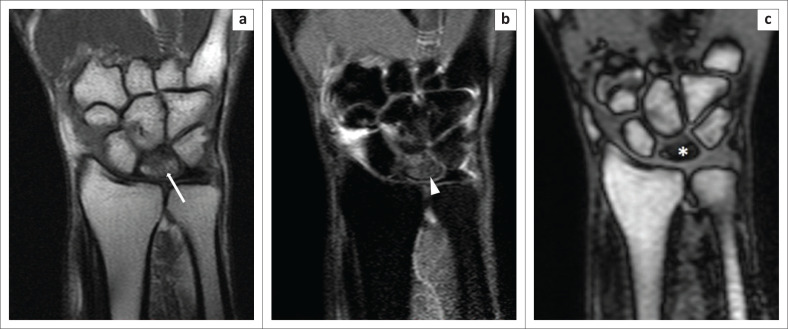
Kienbock’s disease. A 32-year-old man underwent magnetic resonance imaging of the left wrist for the evaluation of chronic pain. The T1W image (a) revealed hypointensity (white arrow) within the lunate; the Proton-Density-Weighted spectral attenuated inversion recovery (b) image indicated hyperintensity (white arrowhead), which was suggestive of marrow oedema. The opposed-phase image (c) revealed a clear flattening of the lunate bone, with the sclerosed bone (white asterisk) demonstrating considerable hypointensity.

The zones of avascular necrosis (AVN) and sclerosis are easily appreciated on OP images with a clear demarcation of the infarcted marrow.^1^ Figure 7 demonstrates how the OP image (Figure 7c) clearly delineates the infarcted marrow in AVN of the right femoral head. Similarly, Figure 8 illustrates the utility of OP imaging (Figure 8c) in demarcation of an infarcted lunate bone in Kienbock’s disease.

### Pigmented villonodular synovitis

**FIGURE 9:**
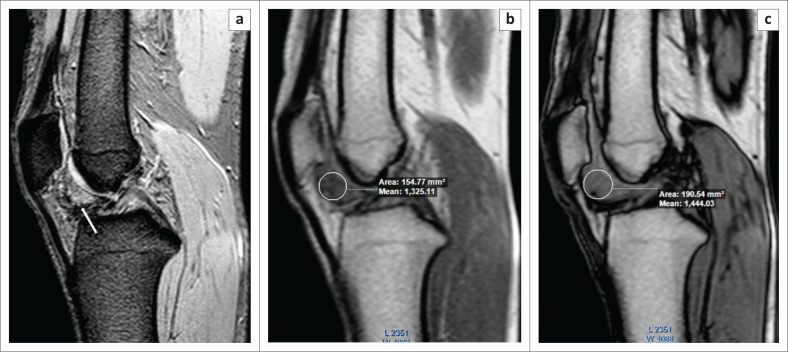
Pigmented villonodular synovitis. A 19-year-old male presented with a history of right knee pain. The T2 GRE (Gradient Echo) image (a) showed hyperintense nodular soft tissue in Hoffa’s fat pad with a few areas of peripheral blooming on the GRE image (white arrow). At chemical shift imaging, the signal intensity of the nodular soft tissue was lower on the in-phase image (b) compared to the opposed-phase image (c), confirming the diagnosis of nodular pigmented villonodular synovitis.

Paramagnetic effects owing to iron accumulation within tissues distort magnetic field microenvironments, with a resultant loss of signal intensity in the affected organs. This signal loss is proportional to the severity of iron deposition and other metallic structures.^18^ In CSI, there is a decrease in signal intensity in the affected tissues on images with longer TE, primarily owing to the T2* decay.^17^ This typically results in increased signal intensity on OP (TE, 2.3 ms) images compared to IP (TE, 4.6 ms) images, which is the opposite of what is observed with fat deposition.^1^ This occurs because the longer TE in the IP sequence allows protons more time to dephase, and hence paramagnetic substances show a decrease in signal intensity in this sequence. Thus, there is an apparent increase in signal on the OP sequence. Hence, the detection of pigmented villonodular synovitis (PVNS) is possible on OP images (Figure 9). Similarly, marrow hemosiderosis, hemarthrosis and haemophiliac arthropathy (hemosiderin deposition in synovium) can be detected on OP images.

## Conclusion

Chemical shift imaging is an important imaging technique that helps to assess various bony pathologies. It aids in detecting and differentiating osseous lesions owing to added tissue contrast without a significant increase in imaging time. Chemical shift imaging could therefore be included as a routine imaging sequence when performing MRI for musculoskeletal disorders.

## References

[1] Pezeshk P, Alian A, Chhabra A. Role of chemical shift and Dixon based techniques in musculoskeletal MR imaging. Eur J Radiol. 2017;94:93–100. 10.1016/j.ejrad.2017.06.011 28655433

[2] Haider MA, Ghai S, Jhaveri K, Lockwood G. Chemical shift MR imaging of hyperattenuating (> 10 HU) adrenal masses: Does it still have a role? Radiology. 2004;231(3):711–716. 10.1148/radiol.2313030676 15118113

[3] Del Grande F, Santini F, Herzka DA, et al. Fat-suppression techniques for 3-T MR imaging of the musculoskeletal system. Radiographics. 2014;34(1):217–233. 10.1148/rg.341135130 24428292 PMC4359893

[4] Bley TA, Wieben O, François CJ, et al. Fat and water magnetic resonance imaging. J Magn Reson Imag. 2010;31(1):4–18. 10.1002/jmri.21895 20027567

[5] Yamashita Y, Torashima M, Hatanaka Y, et al. Value of phase-shift gradient-echo MR imaging in the differentiation of pelvic lesions with high signal intensity at T1-weighted imaging. Radiology. 1994;191(3):759–764. 10.1148/radiology.191.3.8184059 8184059

[6] Mitchell DG, Kim I, Chang TS, et al. Fatty liver: Chemical shift phase-difference and suppression magnetic resonance imaging techniques in animals, phantoms, and humans. Investig Radiol. 1991;26(12):1041–1052. 10.1097/00004424-199112000-00002 1765436

[7] Mitchell DG, Crovello M, Matteucci T, et al. Benign adrenocortical masses: Diagnosis with chemical shift MR imaging. Radiology. 1992;185(2):345–351. 10.1148/radiology.185.2.1410337 1410337

[8] Earls JP, Krinsky GA. Abdominal and pelvic applications of opposed-phase MR imaging. Am J Roentgenol. 1997;169(4):1071–1077. 10.2214/ajr.169.4.9308467 9308467

[9] Disler DG, McCauley TR, Ratner LM, et al. In-phase and out-of-phase MR imaging of bone marrow: Prediction of neoplasia based on the detection of coexistent fat and water. Am J Roentgenol. 1997;169(5):1439–1447. 10.2214/ajr.169.5.9353477 9353477

[10] Delfaut EM, Beltran J, Johnson G, et al. Fat suppression in MR imaging: Techniques and pitfalls. Radiographics. 1999;19(2):373–382. 10.1148/radiographics.19.2.g99mr03373 10194785

[11] Małkiewicz A, Dziedzic M. Bone marrow reconversion–imaging of physiological changes in bone marrow. Pol J Radiol. 2012;77(4):45. 10.12659/PJR.883628 23269936 PMC3529711

[12] Berg BC, Malghem J, Lecouvet FE, et al. Magnetic resonance imaging of the normal bone marrow. Skelprocessetal Radiol. 1998;27(9):471–483. 10.1007/s002560050423 9809875

[13] Del Grande F, Farahani SJ, Carrino JA, et al. Bone marrow lesions: A systematic diagnostic approach. Indian J Radiol Imag. 2014;24(3):279. 10.4103/0971-3026.137049 PMC4126144 25114392

[14] Zajick Jr DC, Morrison WB, Schweitzer ME, et al. Benign and malignant processes: Normal values and differentiation with chemical shift MR imaging in vertebral marrow. Radiology. 2005;237(2):590–596. 10.1148/radiol.2372040990 16244268

[15] Kohl CA, Chivers FS, Lorans R, et al. Accuracy of chemical shift MR imaging in diagnosing indeterminate bone marrow lesions in the pelvis: Review of a single institution’s experience. Skeletal Radiol. 2014;43:1079–1084. 24781818 10.1007/s00256-014-1886-6

[16] Erly WK, Oh ES, Outwater EK. The utility of in-phase/opposed-phase imaging in differentiating malignancy from acute benign compression fractures of the spine. Am J Neuroradiol. 2006;27(6):1183–1188. 16775260 PMC8133913

[17] Eito K, Waka S, Naoko N, Makoto A, et al. Vertebral neoplastic compression fractures: Assessment by dual-phase chemical shift imaging. J Magn Reson Imag. 2004;20(6):1020–1024. 10.1002/jmri.20213 15558548

[18] Westphalen AC, Qayyum A, Yeh BM, et al. Liver fat: Effect of hepatic iron deposition on evaluation with opposed-phase MR imaging. Radiology. 2007;242(2):450–455. 10.1148/radiol.2422052024 17255416

